# OCEAN trial: rethinking long-term anticoagulation after atrial fibrillation ablation

**DOI:** 10.1093/ehjcvp/pvaf084

**Published:** 2025-12-12

**Authors:** Mattia Galli, Beatrice Simeone, Sebastiano Sciarretta

**Affiliations:** Department of Medical-Surgical Sciences and Biotechnologies, Sapienza University of Rome, Corso della Repubblica, 79, Latina 04100, Italy; Maria Cecilia Hospital, GVM Care & Research, Cotignola 48033, Italy; Department of Medical-Surgical Sciences and Biotechnologies, Sapienza University of Rome, Corso della Repubblica, 79, Latina 04100, Italy; Department of Cardiology, ICOT Istituto Marco Pasquali, Latina, Italy; Department of Medical-Surgical Sciences and Biotechnologies, Sapienza University of Rome, Corso della Repubblica, 79, Latina 04100, Italy; IRCCS NeuroMed, Pozzilli, Italy

Catheter ablation is increasingly recognized as an effective treatment option for selected patients with atrial fibrillation (AF), improving both clinical outcomes and quality of life.^1^ Nevertheless, current guidelines recommend continuation of oral anticoagulation (OAC) according to the patient’s CHA_2_DS_2_-VA score (Class I, Level of Evidence C), irrespective of post-procedural rhythm status.^1^ However, long-term OAC increases bleeding risk, making individualized risk–benefit assessment essential. In addition, growing recognition that ischemic risk in AF depends on both clinical factors and AF burden has challenged the need for continued anticoagulation in low-risk patients who remain free of documented atrial arrhythmias after ablation.^2^

On this background, the international, randomized, open-label, *Optimal Anticoagulation for Enhanced Risk Patients Post-Catheter Ablation for Atrial Fibrillation* (OCEAN) compared low-dose aspirin (70–120 mg once daily) with rivaroxaban (15 mg once daily) in 1284 patients with a CHA_2_DS_2_-VASc score ≥1 (≥2 for women) who had undergone AF ablation at least 1 year earlier and had no evidence of recurrent atrial tachyarrhythmias.^3^ Participants were largely low-risk, with a mean age of 66 years and a mean CHA_2_DS_2_-VASc score of 2.2. Most had normal or only mildly enlarged atria and low rates of prior stroke (∼4%), diabetes (∼14%), or coronary artery disease (∼11%). Women were underrepresented (∼28%), and the majority of patients had paroxysmal AF (∼66%). The primary endpoint included stroke, systemic embolism, and new covert cerebral infarcts ≥15 mm detected by magnetic resonance imaging.^3^

At 3 years, the primary composite outcome occurred in 5 patients (0.8%) in the rivaroxaban group and 9 patients (1.4%) in the aspirin group (*P* = 0.28). Fatal or major bleeding occurred in 10 patients (1.6%) vs. 4 patients (0.6%), and the overall rate of major or minor bleeding was higher with rivaroxaban than with aspirin (12.9% vs. 3.6%).^3^ These results must be interpreted in the context of the unexpectedly low incidence of the primary endpoint, reflecting the very low baseline ischemic risk of the study population. Indeed, given the observed event rates, a sample size exceeding 11 000 patients would have been required to adequately power the trial for its primary endpoint. Additional limitations include the absence of a placebo-controlled arm and the use of a rivaroxaban dose (15 mg) that differs from that employed in pivotal randomized trials.

Together with ALONE-AF,^4^ the OCEAN trial strengthens the emerging view that post-ablation ischemic risk is determined by both the patient’s clinical profile and their arrhythmic burden. These findings suggest that, in carefully selected low-risk individuals with persistent sinus rhythm after ablation, the bleeding hazards of long-term OAC may outweigh its potential benefits. Further evidence is needed to refine risk stratification and tailor antithrombotic therapy to each patient’s individualized thrombotic and bleeding profile, as well as their concomitant treatments and lifestyle factors, such as engagement in activities with elevated trauma risk.^5^

## Data Availability

No research data have been used for this manuscript.
